# Sinonasal adenocarcinoma presented as a giant anterior cranial fossa mass: a case report and review of the literature

**DOI:** 10.1186/s13256-024-04413-6

**Published:** 2024-03-04

**Authors:** Endris Hussen Ali, Mulualem Wendafrash Mengesha

**Affiliations:** 1 Department of Neurosurgery, St. Paul’s Millennium Medical College, Addis Ababa, Ethiopia; 2 St. Paul’s Millennium Medical College Hospital, Addis Ababa, Ethiopia

**Keywords:** Sinonasal, Intestinal mucinous type, Adenocarcinoma, Anterior cranial fossa mass

## Abstract

**Background:**

Intestinal adenocarcinoma accounts for less than 0.1–4% of all malignancies in the region. It is common among woodworkers and leather workers. Sinonasal adenocarcinoma usually arises from the ethmoid sinus (40%) or nasal cavity (25%). Extension to nearby structures is common, but intracranial spread is very rare. These tumors are usually treated with surgery, with a reported 5-year survival rate of 59% to 80%.

**Case presentation:**

This is a 60-year-old Black African male patient who presented with globalized headache, nasal obstruction with snoring during sleep, anosmia, change in mentation, sometimes agitation and left-side visual loss of one-year duration with worsening his above symptoms over the last one month. He couldn’t smell soap bilaterally; in his left eye he could see only hand movement at nearly 30 cm. On brain magnetic resonance imaging, there was a T1 hypo- and T2 hyper-intense anterior cranial fossa mass arising from the left ethmoid sinuses and sphenoid sinuses and compressing the left optic structures, and brain computed tomography demonstrated heterogeneous hypo- to isodense mass. Complete tumor excision achieved and discharged with significant improvement and linked to oncology unit for radiotherapy.

**Conclusion:**

The management of these patients is multidisciplinary, involving neurosurgeons, otolaryngologists, oncologists, and maxillofacial surgeons. Surgical resection is the main treatment strategy, followed by radiotherapy, particularly intensity-modulated therapy. Chemotherapy is used in highly advanced, metastatic, and unresectable tumors.

## Introduction

Tumors of the sinonasal area are not common. Different histological types with different grading systems have been mentioned in the literature. Intestinal-type adenocarcinoma is a malignant tumor with invasion of adjacent structures, including the orbit and intracranial extension. It has many similar features to adenomas, carcinomas, or normal intestinal histology. It tends to affect men aged 50–60 years. It is most often found in the ethmoid sinus (40%), followed by the nasal cavity (25%), and the maxillary antrum (20%). The literature has demonstrated long-term exposure to wood dust and the occurrence of intestinal-type adenocarcinoma. Workers with occupational exposure to hardwood dust may show an incidence 1000 times that of the general population. Occupational exposure to wood dust was observed in approximately 20% of workers. Surgery, followed by radiotherapy, is the main management strategy. In the 213-patient study, intestinal-type adenocarcinoma, 50% of patients relapsed, 8% had cervical lymph node metastases, 13% had distant metastases, and 60% died of their disease. Well-differentiated papillary have intestinal-type adenocarcinoma has a good prognosis, but patients with solid and mucinous intestinal-type adenocarcinoma have a poor prognosis [1].

## Case presentation

**Fig. 1:**
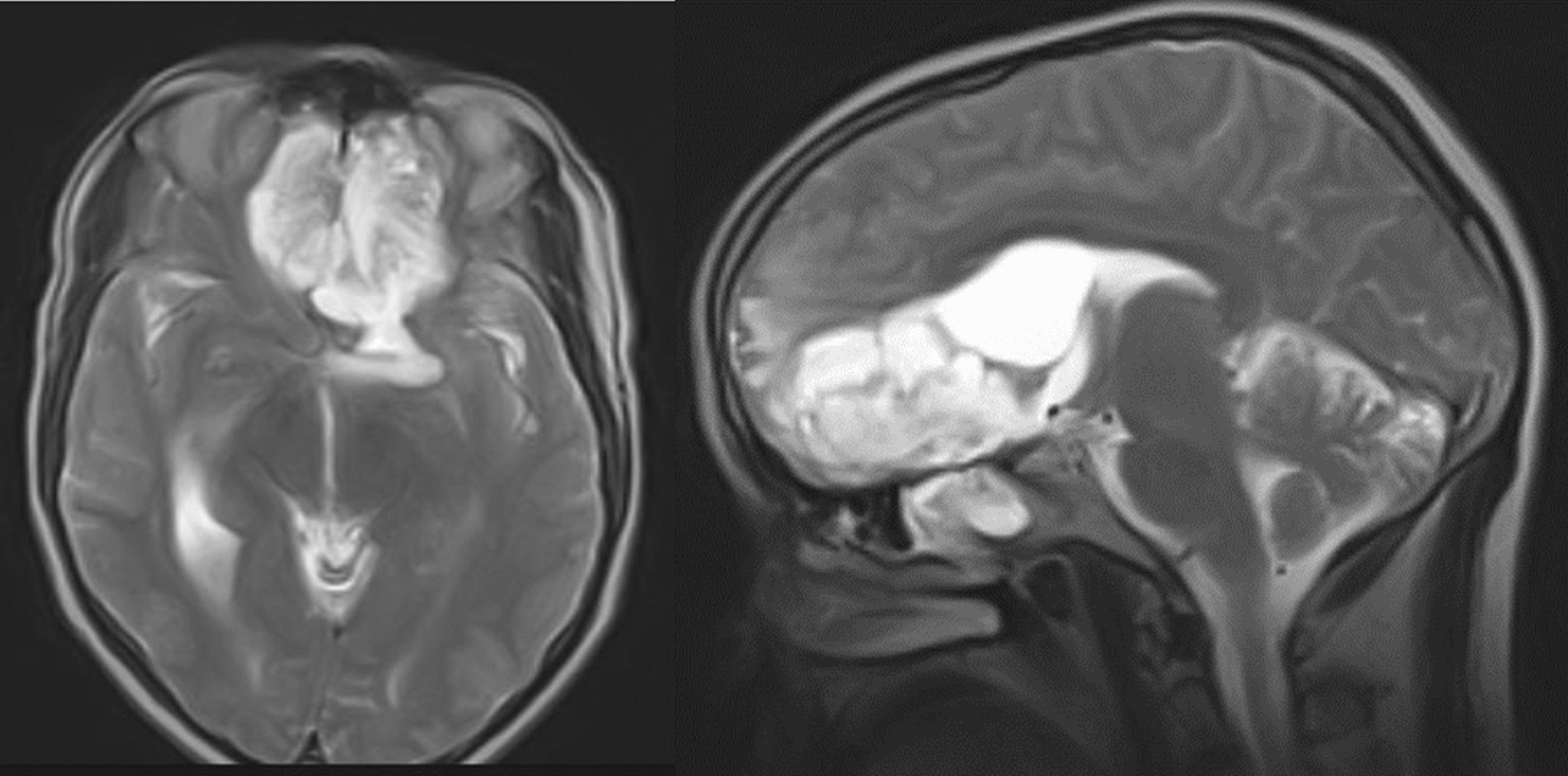
Preoperative T2-weighted images axial and sagittal images demonstrating hyper intense lesion with perilesional edema at olfactory groove region

**Fig. 2:**
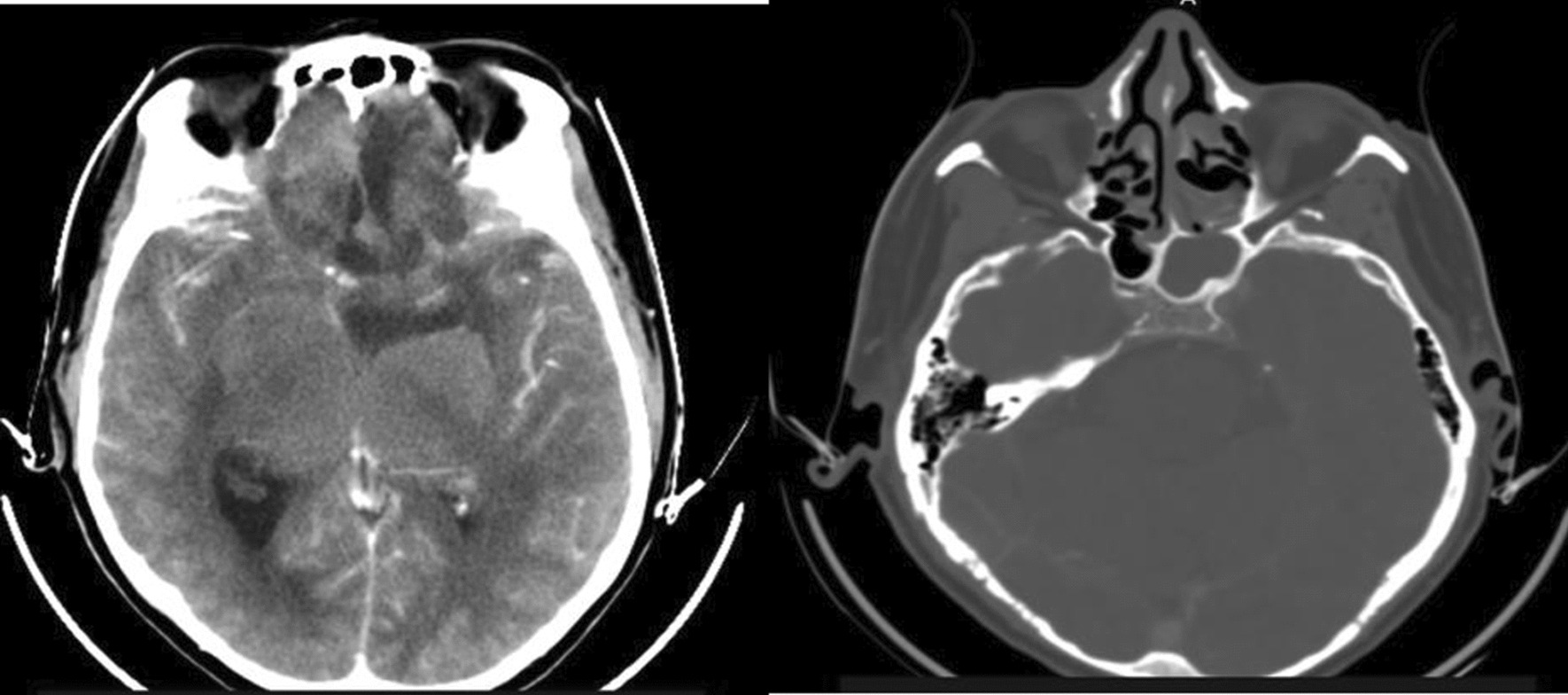
Preoperative precontrast computerized tomography scan demonstrating heterogeneous hypo to isodense lesion at olfactory groove area and bone window demonstrating origin of the tumor from ethmoid and sphenoid opacified sinuses

**Fig. 3:**
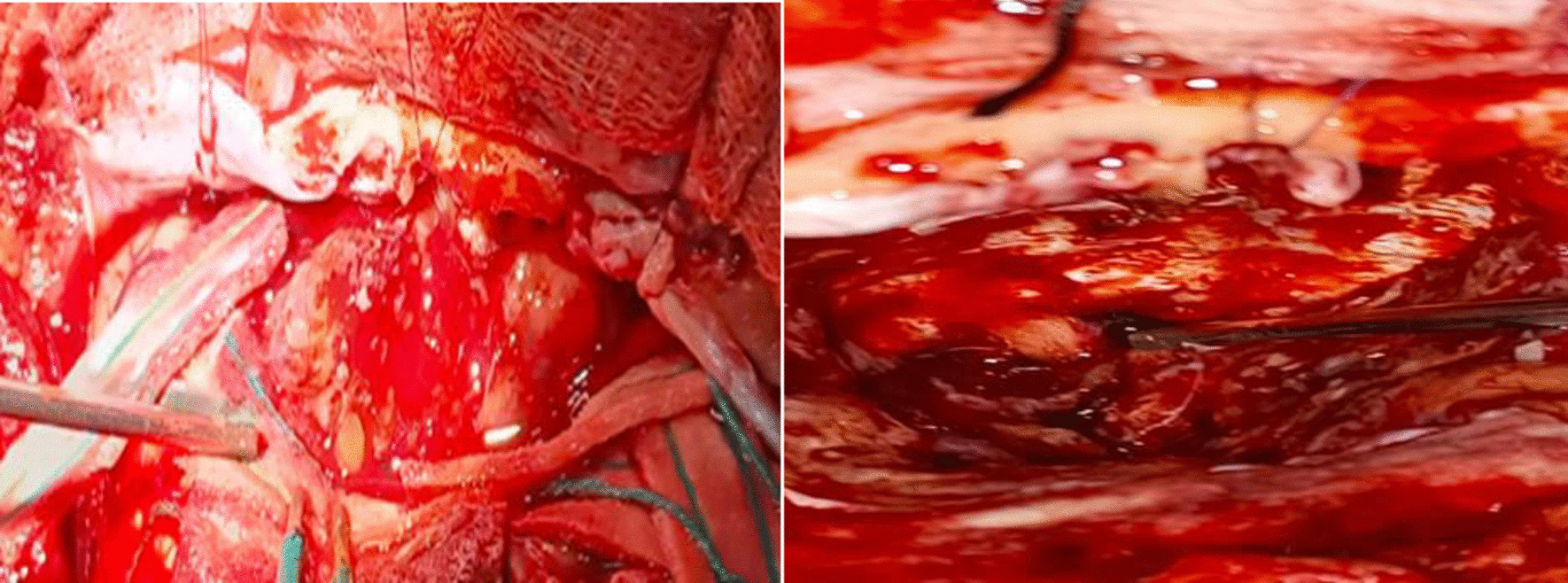
Intraoperative photo demonstrating intradural tumor with extradural extension to ethmoid sinuses and on the second photo after complete resection of the tumor demonstrating optic structures, internal carotid artery and pituitary infundibulum

**Fig. 4:**
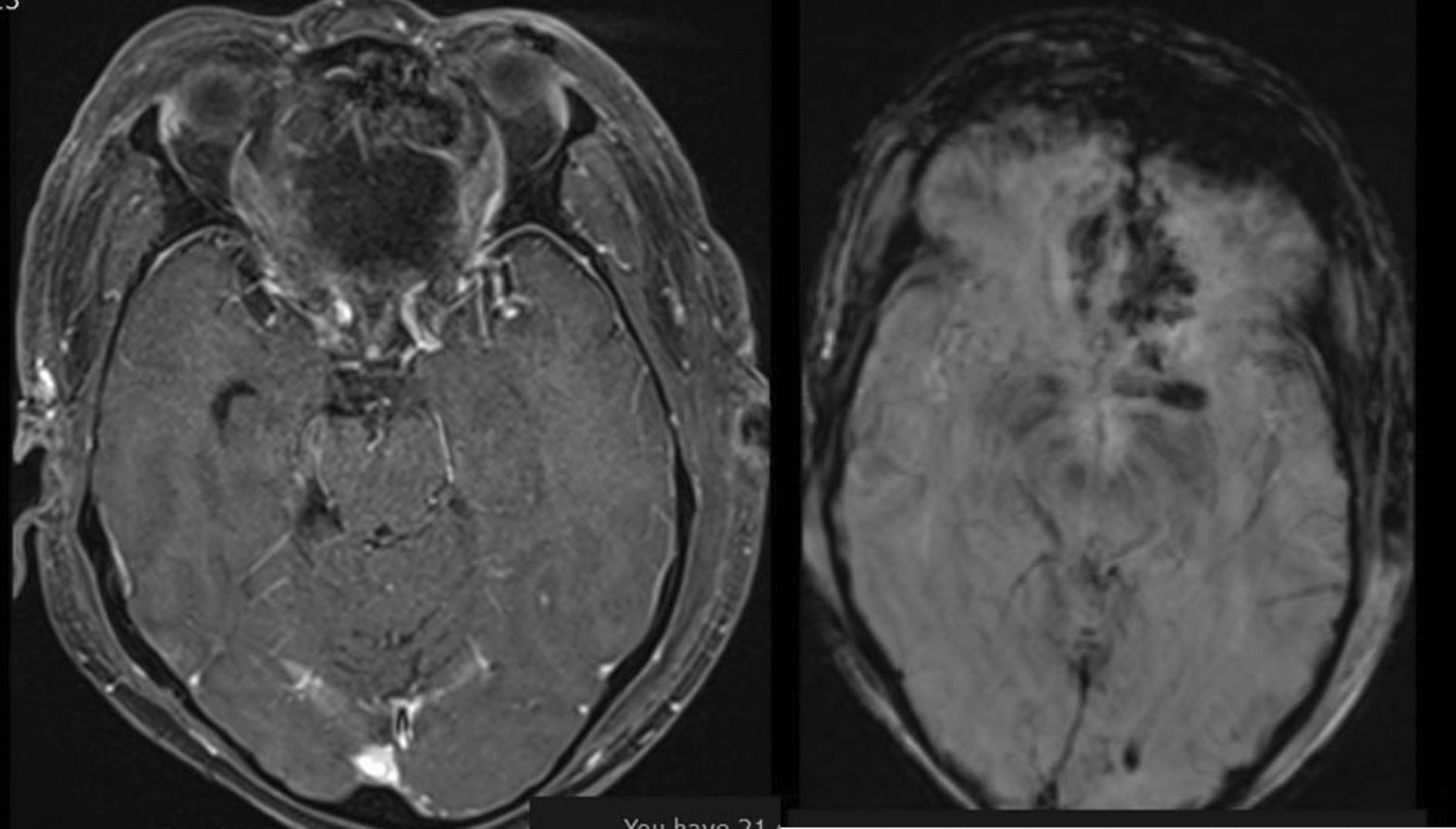
Postoperative post contrast axial magnetic resonance imaging image and susceptibility weighted imaging axial images demonstrating complete resection of the tumor

**Fig. 5:**
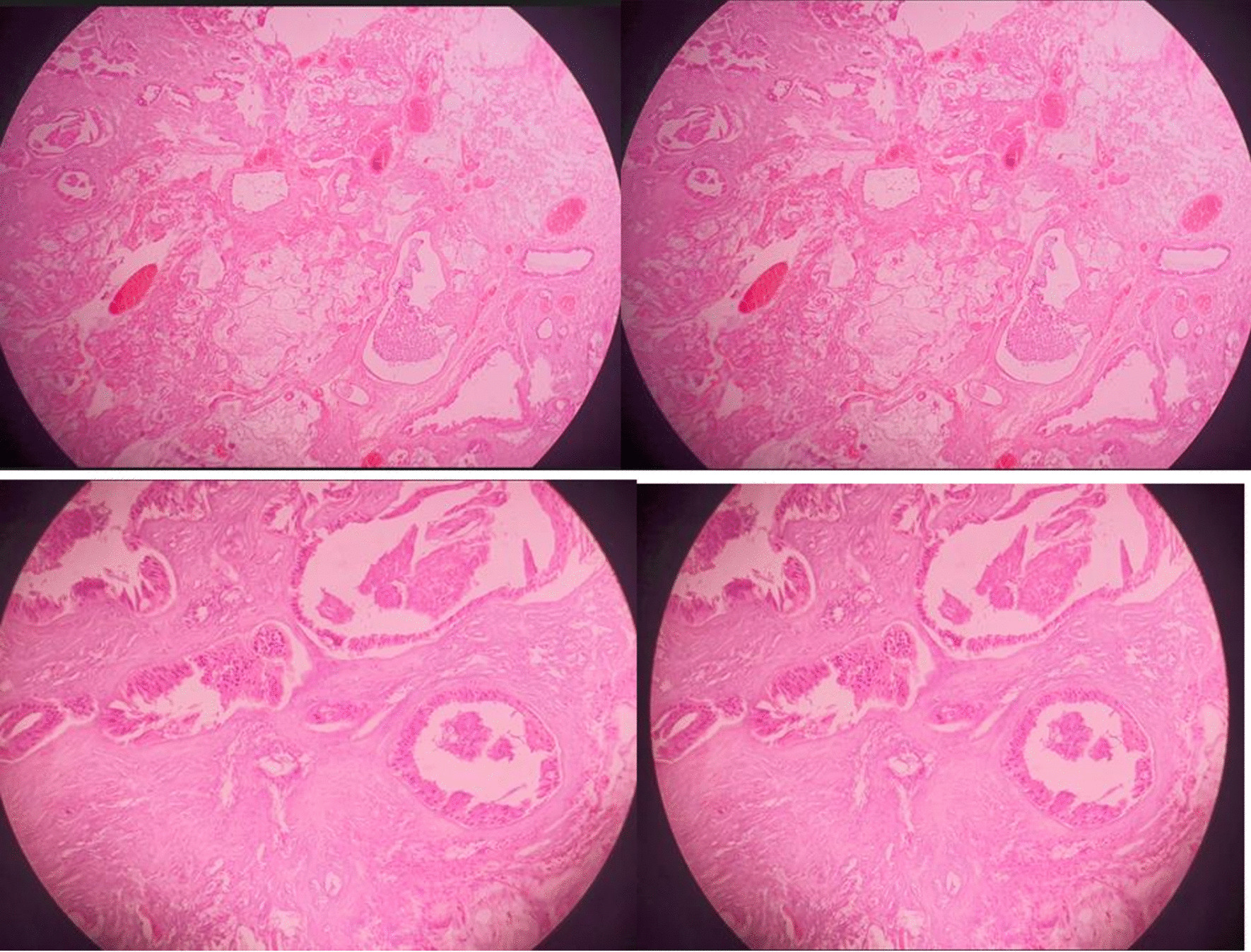
Histopathology microscopic images demonstrating irregular tissue fragments consisting of variable sized glands, lined by single to pseudostratified columnar cells with apical mucinous cytoplasm embedded in a desmoplastic stroma

This is a 60-year-old Black African male patient who presented with globalized headache, nasal obstruction with snoring during sleep, anosmia, change in mentation, sometimes agitation and epistaxis of one-year duration, left-side visual loss that begun one-month before presentation, but no history of exposure to wood dust or leather work, and no smoking history. The mini mental state score was 28/30, the glasgow coma score (GCS) was 15/15, he couldn’t smell soap bilaterally, in his left eye he could see only hand movement at nearly 30 cm, and on his right side he could count from 6 m. Other cranial nerve examinations were unremarkable. Complete blood counts and organ function tests were unremarkable. On brain MRI, there was a T1 hypo intense and T2 hyper intense anterior cranial fossa lesion arising from the left ethmoid sinuses and sphenoid sinuses and compressing the left optic structures, and brain CT demonstrated heterogeneous hypo- to isodense masses (see Figs. 1 and 2).we took him to OR and we did bicoronal skin incision and bifrontal craniotomy done, then extradural DE vascularization of the tumor and dissection plane created between the tumor and frontal lobes, then tumor debulked centrally with suction for the suckable part and with microsissor for the non suckable portion, complete tumor excision achieved and optic nerve, optic chiasm, ICA, pituitary infundibulum identified and protected from any iatrogenic injury(see Fig. 3), patient extubated and transferred smoothly with GCS 15/15,behavioral change was the same as the preoperative baseline on first 3 days then completely resolved on the 6th day, vision on the left side then he can count from 2 m, anosmia remain the same as the preoperative state, no epistaxis, nasal obstruction improved with no night snoring. Postoperative control magnetic resonance imaging (MRI) demonstrated a gross total resection of the tumor (Fig. 4). The biopsy result was irregular tissue fragments consisting of variable-sized glands lined by single to pseudostratified columnar cells with apical mucinous cytoplasm embedded in a desmoplastic stroma, confirming sinonasal intestinal mucinous type adenocarcinoma (see Fig. 5). Then patient discharged and linked to oncology unit for radiotherapy and he is still waiting to get his que as radiotherapy is available in only a few centers in our country.

## Discussion

Malignant neoplasms of the paranasal sinuses are rare, accounting for less than 3% of all head and neck cancers and 1% of all malignancies. Adenocarcinoma accounts for approximately 13% of air sinus malignancies, although there are geographic differences. This type of histopathology is characterized by the non-salivary glandular type and is divided into two types: intestinal adenocarcinoma (ITAC) and non-intestinal adenocarcinoma (NITAC). NITAC is divided into two subgroups: low-grade and high-grade. ITAC can be classified into five subgroups, according to Barnes: the papillary subtype (well-differentiated adenocarcinoma) shows a predominance of papillary structures and the occasional presence of tubular glands. The colon subtype (with moderate differentiation) shows the predominance of glandular-tubular architecture. The solid subtype (poor differentiation) shows loss of differentiation and is characterized by solid and trabecular growth. The slimy subtype includes two growth patterns. One is enlarged glands with mucus and pools of extracellular mucin, known as the alveolar pattern, and the other is solid clusters of signet ring cells, known as the signet ring pattern. A mixed subset consists of a mixture of two or more previously defined patterns [2].

There is a strong association with occupational exposure to wood and leather dust, which usually affects men. The Belgian Cancer Registry documents exposure to various dusts such as wood, fiber, grain, cement, and leather. Between 1978 and 1994, 63% of patients who were diagnosed with sinus adenocarcinoma died [3]. The first occupational exposure to wood dust and the development of adenocarcinoma of the sinonasal region take up to 40 years. Cancers due to occupational exposure have a predominance in men, with 85–95% of these lesions arise in the ethmoid sinuses (see Table 1). Sporadic tumors tend to attack women, and the maxillary antrum is the usual site in 20–50% of cases. Sporadic ITAC tend to have a poor prognosis with short survival. Tumors arising in the maxillary sinus (sporadic cases) remain asymptomatic until they are at an advanced stage, but tumors in the nasal cavity and ethmoid sinus become symptomatic before they invade local structures [4]. Sites for the origin of ITAC were as follows: ethmoid sinuses, 40%; nasal cavity, 28%; maxillary antrum, 23%; and undetermined, 9% [4]. Patients present with various symptoms such as headache, hyposmia, anosmia, nasal obstruction, epistaxis, seizures, confusion, and visual loss (see Table 1). The diagnosis is often made late because these tumors are asymptomatic or produce non-specific symptoms in their early stages.

**Table 1:** Previous sinonasal intestinal type adenocarcinoma cases

Reference number	Authors	Age/sex	Origin	Extension to the brain	Symptoms and duration	Imaging	Surgery	Outcome and complication	Radiotherapy given
3	[7]	55/M	Skull base	None	Carpenter with rapid left visual loss	Skull base and meningeal invasion	Done	Patient died at 10th postoperative week	Given
4	[3]	68/m	Right paranasal sinuses	Extension to both frontal lobes with perilesional edema	Memory loss Confusion 6 months	Large mass involving the right nasal cavity, paranasal sinuses with brain extension	Done	Not mentioned	Not mentioned
5	[8]	56/m	Recurrent change in mentation 6 month postoperative	Extension to both frontal lobes with perilesional edema	Change in mental status 6 months	Large destructive mass in sinonasal area with extension to frontal l; obes, CXR-multiple metastatic lesion in both lungs	Not mentioned	Not mentioned	Not mentioned
6	[4]	68/m	Ethmoid sinuses	High signal frontal mass	Confusion Weight loss 3 month	Frontal lesion hyper intense on magnetic resonance imaging	Frontal craniotomy, orbital osteotomy, tumor resection	Patient developed meningities, discharged at 3rd postoperatively	Not given Due to meningitis
7	[9]	59/m	Nasal cavity from polyp	None	Hyposmia And nasal obstruction 6 months	Complete opacification of all paranasal sinuses	Endoscopic sinonasal surgery, left posterior ethmoidectomy, lateral rhinotomy	Tumor recurrence 2 years later	Radiotherapy given
8	[10]	58/m	Cribriform plate	None	Blurry vision and headache 6 months	Expansile lesion arising from cribriform plate	Tumor resected, lymph node FNAC	Fungal ventriculities, sepsis, deep venous thrombosis	Not given due to unfit patient condition
9	[11]	76/f	Maxillary antrum, ethmoid air cells	None	Nasal obstruction, epistaxis, anosmia 2 months	Soft tissue mass in the maxillary antrum, ethmoid, bone erosion	Tumor resected with craniofacial reconstruction	Unremarkable except for breathing issues which was corrected with stenting	Not given
10	[12]	33/f	Nasal cavity, nasal septum, ethmoid sinus	None	Nasal obstruction 5 months	Nasal cavity, nasal septum, ethmoid sinus	Radical resection with later rhinotomy, small recurrence biopsied	Recurrence biopsied but asymptomatic	Given
11	[13]	72/f	Left nasal cavity	None	Epistaxis and left nasal obstruction	Left middle turbinate mass extending to maxillary sinus, ethmoid sinus, floor of orbit	Left total maxillectomy done-biopsy result low grade adeno carcinoma	Uneventful	Not given

It summarizes the age, sex predominance, tumor origin, presence or absence of extension to the brain, how patients present, duration of their illness, how they appear on brain computerized tomography scan and magnetic resonance imaging, types of surgery done and the outcome after surgery and complications, if radiotherapy was given or not and its effect on patient outcome

*FNAC* fine needle aspiration

Nasal endoscopy helps us know the location and extent of the tumor. Computed tomography (CT) and MRI can tell the exact location and extent of the disease. On MRI, tumors with well-represented mucin content have hyper intensity on T2-weighted images, while ITACs without mucin show isointensity to hypo intensity on T2-weighted images [5] (see Table 1).

In many cases, both CT and MRI are necessary for accuracy and the treatment plan. After imaging, an endoscopic biopsy of the lesion is a must to clearly determine the specific histological type of the tumor. Tumors with aggressive histological types need a repeat biopsy. A complete staging of the patient is mandatory. An ultra-sonographic examination of the neck and a contrast CT examination of the chest and abdomen are performed to rule out regional or systemic metastasis. A whole-body PET-CT examination is used in aggressive histological types such as sarcoma, malignant melanoma, undifferentiated lesions, and advanced-stage lesions [5]. Carcino-embryonic antigen (CEA) has been used to differentiate metastatic colon adenocarcinomas from primary ITAC, with strongly positive staining supporting metastatic disease [3].

Surgical resection is the cornerstone of the treatment of such cancers. Endoscopic endonasal resection is an effective treatment strategy alone for early-stage (T1–T2) low-grade lesions. Radially resected lesions with negative margins are the goal of the surgery [5].

Although endoscopic resection is found to be as effective as open surgery in skull base lesions, in our case, open surgical resection was a must as the tumor was not accessible for endoscopic resection by otolaryngologists tried to access the tumor endoscopically by trans nasal route 1 month before we operate him, however it was difficult to identify the tumor. Hence, we did open surgical resection in a Supine position with a slightly extended neck, a bicoronal skin incision with a bifrontal craniotomy, and then extradural cauterization. The anterior ethmoidal arteries and posterior ethmoidal arteries are the main feeders; hence, they must be identified, cauterized, and divided to decrease intraoperative blood loss. The crista galli is carefully separated from the dura mater and removed with rongeured while keeping the dura intact. The key point for the subsequent optimal reconstruction of the skull base is to properly dissect the epidural space above the orbital roofs laterally, the planum sphenoidale posteriorly, and the back wall of the frontal sinus anteriorly. The dura opened in a c-shaped fashion starting at the lateral ends of the tumor extending to the midline. The falx cerebri is cut on the anterior third of the sagittal sinus after the sagittal sinus was ligated. the tumor debulked centrally, rolling tumor edges to the center, with a special precaution to protect the ICA, ACA, optic nerve, oculomotor nerve, and pituitary stack during tumor resection at its base, where these structures are intimately related and located [5].

The dural defect is reconstructed with primary dural repair, with a few defects closed with duraplasty and pericranial grafts pedicled to add more layers to prevent CSF leaks and their complications (see Table 1).

For most patients with well-developed frontal sinus cranialization needs to done that is the inner table of the frontal sinus has to be rongeured, the mucosa of the sinus exenterated, and the fronto-nasal duct should be packed with muscle to prevent mucocele formation and surgical site infections; Including epidural abscess, subdural abscess, brain abscess, osteomyelitis, and cranioplasty material infection, if used.

surgical resection is adequate for most low stage (pT1–2) and low-grade ITACs (papillary, colonic). In this case, there is no need to consider radiotherapy. In patients (pT3–4, solid/mucinous subtypes, and/or positive margins intensity-modulated radiation therapy is necessary. Post-operative IMRT is recommended for high-grade sinonasal adenocarcinomas (G3, signet-ring variant, solid type). Adjuvant IMRT is used in advanced-stage lesions (T3–T4) and in the presence of positive surgical margins. Maximum dose adjuvant radiotherapy ranges from 50–70 Gy, depending on risk factors, and is given in fractions of 1.8–2 Gy. A neck lymph node biopsy is not routinely performed for sinonasal adenocarcinoma due to the low risk of regional metastases (7%). The need to include the level of the neck in the postoperative radiation field is more thoughtful as involvement of the neck is low, except for advanced T stages. There is no strong evidence for the use of adjuvant concurrent chemo -radiation for ITAC [6].

In advanced-stage ITAC (T3–T4), a chemotherapy regimen composed of cisplatin, fluorouracil, and leucovorin for tumors with non-mutated-wild-type p53 tumor suppressor gene combining them with surgery and radiation is found to improve survival rates [5]. This chemotherapy regimen can be used in the palliative care of patients with symptomatic, unresectable, and/or metastatic tumors. Local application of 5-fluorouracil once or twice a week for 4–6 weeks after surgery for ITAC with a good result is reported [6].

Immune therapy might be implemented in the multidisciplinary treatment of selected ITACs. The immunotherapeutic potentials of ITAC remain theoretical and not clinical [6].

The problem of treatment failure with advanced disease is high. Franchi *et al*. reviewed the outcomes of 41 ITAC patients followed for more than 108 months. About 46% of tumors recurred, and 56% died. Patients with less extensive disease who are candidates for complete surgical resection with or without postoperative radiation showed better results. These patients are sometimes cured [3] (see Table 1).

In accordance with the “European Position Paper on Endoscopic Management of Tumors of the Nose, Paranasal Sinus, and Skull Base,” follow-up of treated patients for ITAC should be with endoscopic evaluation and MRI every 2–3 months on the first year, every 6 months for 2–5 years, then once per year for 10 years. Although not based on fixed evidence, lifelong follow-up is generally recommended [6].

## Conclusion

Endoscopic evaluation, brain MRI and brain CT scan, carcinoembryonic antigen test, metastasis workup with abdominopelvic and chest CT scan, and neck ultrasound for lymph nodes are recommended. The management of these patients is multidisciplinary, involving neurosurgeons, otolaryngologists, oncologists, and maxillofacial surgeons. Surgical resection is the main treatment modality, followed by radiotherapy, particularly intensity-modulated therapy. Chemotherapy is used in highly advanced, metastatic, and unresectable tumors. Mucinous and solid type sinonasal adenocarcinoma has poor prognosis. Immunotherapy is not clinically applicable yet. Despite the treatment, the recurrence rate remains very high.

## Data Availability

Not applicable.

## Abbreviations

CSF: Cerebrospinal fluid
CT: Computerized tomography
MRI: Magnetic resonance imaging
T1W: T1-weighted images
T2W: T2-weighted images
DWI: Diffusion-weighted images
ITAC: Intestinal type adenocarcinoma
Gy: Gray
IMRT: Intensity modulated radiotherapy
PET-CT: Positron emission tomography scan
NITAC: Non-intestinal adenocarcinoma
CEA: Carcinoembryonic antigen
NEC: Neuroendocrine carcinoma
GCS: Glasgow coma score
Adcc: Adenoid cystic carcinoma
ICA: Internal carotid artery
ACA: Anterior cerebral artery
OR: Operating room
DVT: Deep venous thrombosis
